# Comparative transcriptome analysis reveals a serotype-specific immune response in Nile tilapia (*Oreochromis niloticus*) infected with *Streptococcus agalactiae*

**DOI:** 10.3389/fimmu.2024.1528721

**Published:** 2025-01-10

**Authors:** Renan José Casarotto Appel, Karine Nicole Siqueira, Ioannis Konstantinidis, Maria Isabel Mello Martins, Rajesh Joshi, Lucienne Garcia Pretto-Giordano, Laurival Antônio Vilas-Boas, Jorge Manuel de Oliveira Fernandes

**Affiliations:** ^1^ Faculty of Biosciences and Aquaculture, Nord University, Bodø, Norway; ^2^ Department of General Biology, State University of Londrina, Londrina, Brazil; ^3^ Department of Veterinary Clinics, State University of Londrina, Londrina, Brazil; ^4^ GenoMar Genetics AS, Oslo, Norway; ^5^ Department of Preventive Veterinary Medicine, State University of Londrina, Londrina, Brazil; ^6^ Department of Renewable Marine Resources, Institute of Marine Sciences (ICM-CSIC), Barcelona, Spain

**Keywords:** Nile tilapia, brain, streptococcosis, immunity, aquaculture, gene expression, RNAseq

## Abstract

*Streptococcus agalactiae* is a major causative agent of streptococcosis in Nile tilapia (*Oreochromis niloticus*) and understanding its etiology is important to ensure the sustainable development of global tilapia farming. Our research group recently observed contrasting disease patterns in animals infected with two different *S. agalactiae* serotypes (Ib and III). To better understand the basis for these divergent responses, we analyzed the brain transcriptome of Nile tilapia following bacterial exposure. Our findings revealed significant variation in the expression of genes involved in immune (e.g., *CD209* antigen, *granulin*, C-X-C motif chemokine 10, prostacyclin synthase, and interleukins) and neuroendocrine (e.g., *mmp13a*, *mmp9*, *brain aromatase*, and *pmch*) pathways. The serotype Ib strain seems promptly recognized by the host, triggering a potent inflammatory response, whereas the serotype III strain elicited a less immediate response, resulting in more pronounced central nervous system (CNS) symptoms and behavioral effects. To the best of our knowledge, this is the first study to show serotype-specific immune responses to *S. agalactiae* in Nile tilapia. These findings are important for advancing disease management and control strategies in aquaculture. Identifying different immune reactions triggered by serotypes Ib and III may assist the development of more specific approaches for preventive measures, early detection, and effective treatment against streptococcosis.

## 1 Introduction

The aquaculture industry is continuously evolving towards more intensive cultivation systems. Although this shift has led to a production increase, it has also promoted the proliferation of opportunistic and pathogenic bacteria (1). Tilapia farming has become one of the most important industries in the aquaculture sector (2), making Nile tilapia (*Oreochromis niloticus*) the second-most cultivated fish globally (1, 3). Rising consumer demand for high-quality animal protein has led to a diversification in the tilapia production systems, with the farms now ranging from conventional small-scale culture to intensive high-density stocking systems (4). However, this intensification resulted in a significant challenge: disease outbreaks. High stocking densities and consequent poor water quality create stressful conditions that suppress the fish immune system, making them more vulnerable to infections and facilitating disease transmission.

Among bacterial diseases in tilapia, streptococcosis causes significant economic losses for producers worldwide (5, 6). *Streptococcus agalactiae*, a Gram-positive encapsulated bacterium, is the main pathogen associated with streptococcosis (7–9). The disease manifests with severe clinical signs, such as loss of appetite, scoliosis, ascites, exophthalmia, corneal opacity, and meningoencephalitis, leading to neurological symptoms like disorientation and erratic swimming (10, 11). Current strategies to combat streptococcosis include vaccination, antibiotics, and selective breeding for genetic resistance (2, 12). However, the diversity of *S. agalactiae* serotypes makes disease control challenging, with isolates showing unique pathogenic profiles and treatment responses (13–16). *S. agalactiae* is classified into ten serotypes (Ia, Ib, II, III, IV, V, VI, VII, VIII, and IX) based on capsular antigens (17), with serotypes Ia, Ib, and III being the most common in fish worldwide (18).

Despite extensive research on the overall development of streptococcosis, including the effects of temperature (19–21) and diet (22–25) on disease progression, gene expression variance within fish (26–29), and pathogen immune evasion mechanisms (30–32), our understanding of the divergent infection symptoms and host immune responses to different bacterial serotypes remains limited.

Our research group recently isolated two different serotypes of *S. agalactiae* following streptococcosis outbreaks in Brazil: SA8-UEL (serotype Ib), isolated in Paraná state, and SA10-UEL (serotype III) from Maranhão. After further investigation, it was possible to notice different infection response patterns in Nile tilapia exposed to these strains, with the SA10-UEL strain inducing severe neurological symptoms, including erratic swimming and a high mortality rate in the initial days of infection. In contrast, the SA8-UEL strain exhibited slower disease progression with less pronounced brain-related symptoms (personal observations, unpublished).

Understanding how distinct serotype infections influence immune pathways, blood-brain barrier (BBB) integrity, and neuroendocrine functions is indispensable for predicting and managing streptococcosis dynamics. To address this knowledge gap, we conducted a transcriptome analysis to characterize the brain response in Nile tilapia following exposure to two *S. agalactiae* strains of different serotypes.

## 2 Materials and methods

### 2.1 Ethics statement

All experiments in this study were conducted in strict accordance with the guidelines established by the Animal Ethics Committee of the State University of Londrina, Brazil (process n° CEUA 45/2017).

### 2.2 Fish rearing

Male Nile tilapia (average weight 20 ± 2 g) were obtained from the Vivenda Flora Tropical fingerling fish farm at Londrina, Paraná, Brazil. The fish were acclimated in a 1000 L tank for 15 days and fed twice a day with extruded commercial feed (3% body weight) manufactured by Integrada Agricultural Cooperative, Brazil. Water temperature was kept at 28.0 ± 0.5°C using heating rods (model RS-300, RS Electrical), with a pH range of 6.8–7.2. Dissolved oxygen levels ranged from 7 to 8 ppm, and the photoperiod was maintained at a 14:10-h light:dark schedule.

### 2.3 *S. agalactiae* strains

The two strains of *S. agalactiae*, SA8-UEL (serotype Ib) (accession number SUB14593605) and SA10-UEL (serotype III) (accession number SUB14593588), were cultured in liquid tryptic soy broth (TSB) (Acumedia) at 30 °C overnight without agitation. The cultures were subcultured into 100 mL TSB broth following the same conditions for 8 h. Bacterial concentration was determined in colony-forming units (CFU) per mL by plating 10 μL of 10-fold serial dilutions on tryptic soy agar (TSA) (Acumedia) plates (33).

### 2.4 Fish challenge and sample collection

Prior to the experiment, we randomly selected 1% of the animals received for pre-screening. These fish were anesthetized and euthanized, and their kidneys were collected for plating on selective media and PCR analysis by amplification of the 16S rRNA gene using the F1 (5’-GAG TTT GAT CAT GGC TCA G-3’) and Imod (5’-ACC AAC ATG TGT TAA TTA CTC-3’) primers. Both tests confirmed that the animals were not infected with *S. agalactiae* before the formal challenge experiment. After a 24-hour fasting period, 260 Nile tilapia were randomly divided into three experimental groups: a control group of 20 fish, and two challenged groups with six replicate tanks containing 20 fish each for the SA8-UEL and SA10-UEL strains. Fish were housed in 50 L aquaria. Before bacterial inoculation, the fish were anesthetized with eugenol (100 mg/L) for 30-40 s and intraperitoneally injected with 0.1 mL/fish of bacterial suspension (8 × 10^7^ CFU/mL for SA8-UEL, and 4 × 10^7^ CFU/mL for SA10-UEL). In contrast, control group fish were injected with an equal volume of TSB broth (Acumedia). The bacterial concentrations were selected based on prior experiments to identify minimum concentrations that induce neurological symptoms, such as erratic swimming, in approximately 20% of the animals challenged with *S. agalactiae*. After inoculation, the animals were maintained in a freshwater flow-through system following the same conditions as during the acclimation period.

After bacterial exposure, the fish were fed and monitored daily over the next 15 days for the development of clinical symptoms. The primary objective of the study was to evaluate gene expression in brain tissue after the appearance of erratic swimming behavior, a sign indicative of central nervous system (CNS) damage. Fish were examined daily and those exhibiting such clinical symptoms were promptly removed from the aquaria, euthanized by medullary section, and their brain was collected; a counterpart from the control group was also sampled, except for one SA8-UEL and SA10-UEL strains. The brain tissue was collected immediately after euthanasia by performing a cranial cut with a sterile scalpel and removing the brain using a sterile clamp. The brain was placed in cryogenic tubes containing 1.5 mL of RNAlater (Sigma-Aldrich) and immediately frozen in liquid nitrogen. All time points for sample collection are detailed in **Supplementary Table 1**.

### 2.5 RNA extraction, library construction, and sequencing

Frozen brain samples were homogenized in QIAzol lysis reagent (Qiagen) at 5,000 × g for 20 s with zirconium oxide beads (1.4 mm; Precellys) in a Precellys^®^ 24 homogenizer (Bertin Instruments). RNA was extracted using Direct-zol™ RNA MiniPrep (Zymoresearch) following the manufacturer’s instructions. RNA concentration, purity, and quality were assessed using NanoDrop™ 1,000 (Thermo Fisher Scientific) and Tape Station 4150 (Agilent Technologies). RNA-seq libraries were prepared using the NEBNext Ultra™ RNA Library Prep Kit (New England Biolabs) with the poly(A) mRNA magnetic isolation module (NEB #E7490). Briefly, after poly(A) enrichment of 0.8 ng of total RNA, mRNA was fragmented to ~100–200 nt, before synthesis of the first- and second cDNA strands. The resulting cDNA was purified, end-repaired, and used for adaptor ligation followed by barcoding using NEBNext Multiplex Oligos (New England Biolabs). PCR enrichment was done with 9 cycles, and the amplified libraries were purified using AMPure XP beads (Beckman Coulter, Inc.). In total, 14 libraries were prepared (4 for Control, 5 for SA8-UEL, and 5 for SA10-UEL groups). Libraries were quantified on the Tape Station 4150 (Agilent Technologies), pooled at equimolar ratios, and sequenced as paired-end reads (150 bp) on an Illumina NovaSeq 6000 sequencer (Illumina) at Novogene. The datasets generated in this study are available at the NCBI BioProject database under accession number PRJNA1049341.

### 2.6 Bioinformatic analysis

Adapter sequences and low-quality reads (quality < 20) were removed from the raw reads using *fastp* software (34) (version 0.23.2). Quality-trimmed reads were mapped to the Nile tilapia genome (accession number MKQE00000000.2) downloaded from NCBI (https://ftp.ncbi.nlm.nih.gov/), using HISAT2 (35) (version 2.2.1), and reads were annotated using *featureCounts* (36) (version 2.0.3). Differential expression of genes across treatment groups was analyzed using the R package limma (37), with the criteria |Log2 fold change| ≥ 1 and adjusted p-value of ≤ 0.05 (Benjamini–Hochberg multiple test correction method). Using the R pheatmap package, a heatmap was generated to visualize the fold change values of selected transcripts across different conditions. Genes were categorized into functional groups based on their biological roles, and comparisons with nonsignificant log2 fold change values were treated as zero. Row clustering was performed using hierarchical clustering with Euclidean distance and average linkage method. Enrichment of KEGG pathways and gene ontology (GO) was performed in g:Profiler (38) with a significance threshold of 0.05 (g:SCS multiple test correction method). The packages ggplot2 and GOplot in R were employed to visualize the data.

### 2.7 Fish challenge for biological validation

Following the same experimental setup from the first challenge, we conducted a new experiment to validate our initial transcriptomic findings. The strains of *S. agalactiae* were cultured in TSB (Acumedia) and incubated at 30°C for 18 hours. Subsequently, bacterial cells were collected by centrifugation at 10,000 × g for 20 minutes at 4°C. The bacterial pellets were washed twice with 0.85% saline solution, employing the same centrifugation parameters, and resuspended. The optical density (OD) from each suspension was measured at 620 nm using a spectrophotometer. The OD for the strain SA8-UEL was adjusted to 0.79 and for the SA10-UEL strain to 0.85, to achieve a final bacterial concentration of approximately 1 × 10^9^ CFU/mL for each strain.

In this trial, fish from the challenged groups were intraperitoneally injected with 0.1 mL of the respective bacterial suspension (1 × 10^8^ CFU/mL). This dosage is slightly higher than in the original experiment (see section 2.4) to increase the incidence of neurological symptoms to 25% of challenged animals. Fish from the control group were injected with an equal volume of sterile saline (0.85%). Fish exhibiting erratic swimming behavior were euthanized and 21 brain samples (7 from each group) were collected.

### 2.8 Gene expression analysis by qRT-PCR

The transcriptomic results and expression analysis of ten genes (**Supplementary Table 2**) were validated by qRT-PCR using samples obtained from the biological validation study. Primers for the selected genes were designed using the PrimerQuest™ tool from IDT (Integrated DNA Technologies) (https://eu.idtdna.com/PrimerQuest/). Primer secondary structures and dimers were assessed with NetPrimer (Premier Biosoft). The primers for reference and target genes are given in **Supplementary Table 2**.

Briefly, 1 µg of total RNA extracted from each sample as mentioned previously, was reverse transcribed using the QuantiTect Reverse Transcription kit (Qiagen), according to the manufacturer’s instructions. The obtained cDNA was diluted 50 times with nuclease-free water and used as a PCR template. The PCR reactions to validate the primers were carried out using the AmpliTaq Gold™ 360 Master Mix (Applied Biosystems™), on a C1000 Touch™ thermal cycler (Bio-Rad) programmed as follows: 95°C for 3 min, 40 cycles of 95°C for 30 s, 58-62°C for 34 s, and 72°C for 1 min, followed by another cycle of 72°C for 5 min. PCR products were analyzed by electrophoresis on a 1% (w/v) agarose gel, stained with SYBR Safe (Invitrogen™), and visualized in NuGenius Gel Documentation System (Syngene™) with the O’GeneRuler Express DNA Ladder 5 kb (Thermo Fisher Scientific). Water (negative control) and Nile tilapia DNA (positive control) were used as controls for the PCR reactions. After confirming the correct size of the amplicons, we carried out the qPCR.

The qPCR reactions were conducted using the SYBR Green qPCR Master Mix (Thermo Fisher Scientific) on a CFX96™ Real-Time PCR System (Bio-Rad) programmed as follows: 95°C for 3 min, 40 cycles of 95°C for 30 s, 58-62°C for 34 s, and 72°C for 1 min, followed by a melting curve analysis. The reactions were performed in duplicates of 7 biological replicates in each group. Data were acquired and analyzed using the CFX Maestro software (Bio-Rad).

Using geNorm (39) a geometric normalization factor was computed for each of the samples based on the relative quantities of the two most stable genes (tubulin alpha chain-like (*tuba*) and ubiquitin-conjugating enzyme (*ubce*)) among the set of four reference genes tested, which also included elongation factor 1-alpha (*eef1a*) and beta-2-microglobulin (*b2m*) (40). The relative expression levels were calculated relative to the normalization factors using the 2^−ΔΔCt^ method (41), taking the efficiency of each reaction into account.

For the correlation between RNA-Seq and qPCR data, the average Log2 fold change was calculated for each group within both datasets. These average fold changes were then compared between the infected groups (SA8-UEL and SA10-UEL) and the control group. The significance of differences between the RNA-Seq and qPCR data was analyzed using the Mann-Whitney U Test (Wilcoxon rank-sum test) (p < 0.05). All results are presented in **Supplementary Figure 1** as mean ± standard error.

### 2.9 Detection of *S. agalactiae* in brain tissue by qPCR

To confirm bacterial presence within brain tissue, we performed qPCR targeting the *S. agalactiae rpoB* gene, which encodes the RNA polymerase beta subunit. The analysis utilized cDNA synthesized from samples obtained during the biological validation study. Amplification was carried out using the primers rpoB_F1 (5’-CACAATTCATGGACCAACACAAC-3’) and rpoB_R1 (5’-GGCGTTTGTGCGACAATTCT-3’) (42). The qPCR followed the same parameters as those used in the gene expression assays, with a 60°C annealing temperature.

## 3 Results

### 3.1 RNA sequencing analysis

**Figure 1:**
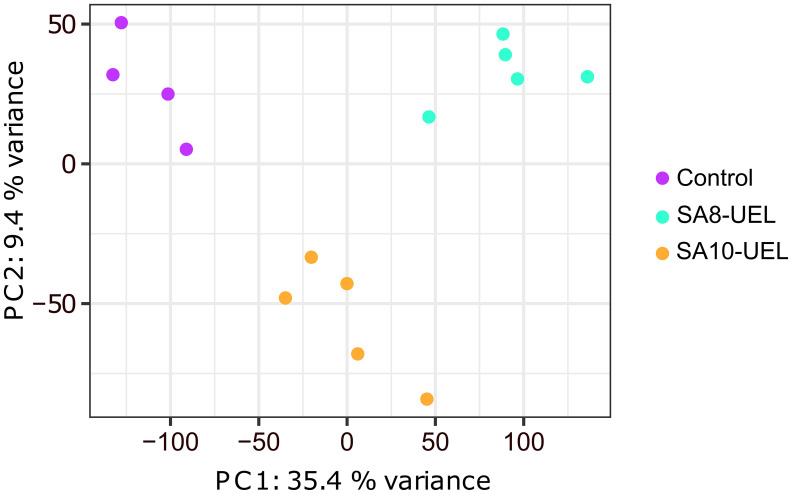
Principal component analysis plot shows the clear clustering of Control, SA8-UEL, and SA10-UEL groups.

A total of 306,649,706 raw reads were obtained from 14 libraries (4 from the Control group, 5 from the SA8-UEL strain exposed group, and 5 from the SA10-UEL strain exposed group), with a range of 15,272,516 to 26,377,837 per library. After adapter trimming and quality filtering, 303,492,279 reads were obtained and 277,440,575 reads were mapped to the reference genome, with an overall mapping rate of 91.3% (**Table 1**). Principal component analyses (PCA) revealed the differential clustering of the SA8-UEL, SA10-UEL, and Control groups across the first principal component (PC1), accounting for 35.4% of data variability (**Figure 1**).

**Table 1:** Summary of assembly statistics for the transcripts obtained from *S. agalactiae* exposed (SA8-UEL or SA10-UEL strains) and control Nile tilapia brain.

Group	Sample ID	Raw read number	Clean read number	Total mapped (%)*
Control	TIL01008	18824055	18676959	90.7
Control	TIL01009	22353288	22231832	89.5
Control	TIL01010	23150678	23047138	89.0
Control	TIL01011	24891027	24466305	91.2
SA8-UEL	TIL01111	19522409	19010902	91.1
SA8-UEL	TIL01112	22260859	22056378	91.6
SA8-UEL	TIL01113	22170619	21834436	92.1
SA8-UEL	TIL01114	21524533	21380984	93.9
SA8-UEL	TIL01115	23498111	23358731	89.9
SA10-UEL	TIL01210	23641051	23502210	90.5
SA10-UEL	TIL01212	23281400	22945481	93.3
SA10-UEL	TIL01214	15272516	15089281	93.5
SA10-UEL	TIL01215	26377837	26130739	92.5
SA10-UEL	TIL01216	19881323	19760903	90.1

*Percentage of the reads mapped to the reference genome.

### 3.2 Differential gene expression analysis

**Figure 2:**
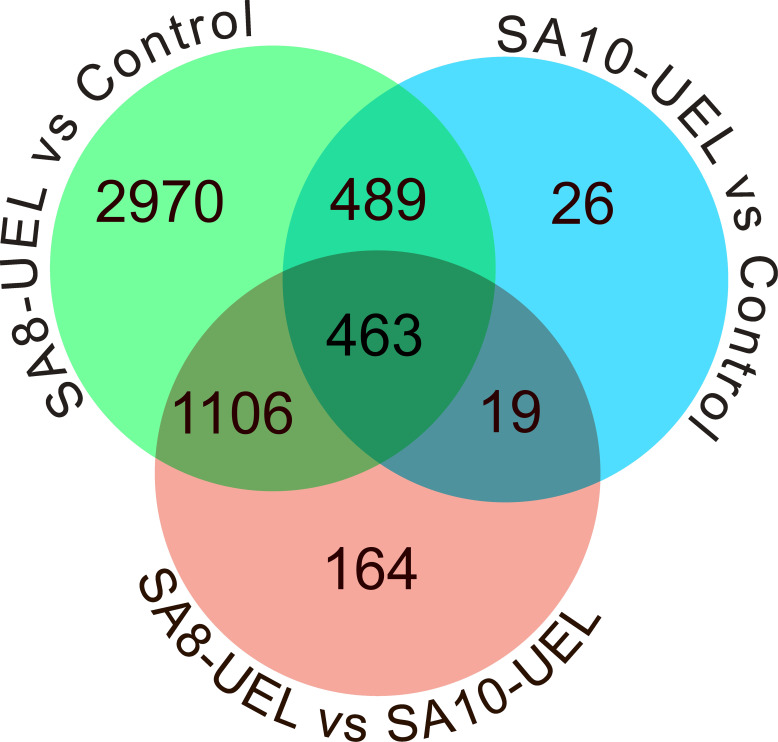
Venn diagram showing the distribution of differentially expressed genes among the comparisons between Control, SA8-UEL, and SA10-UEL groups.

**Figure 3:**
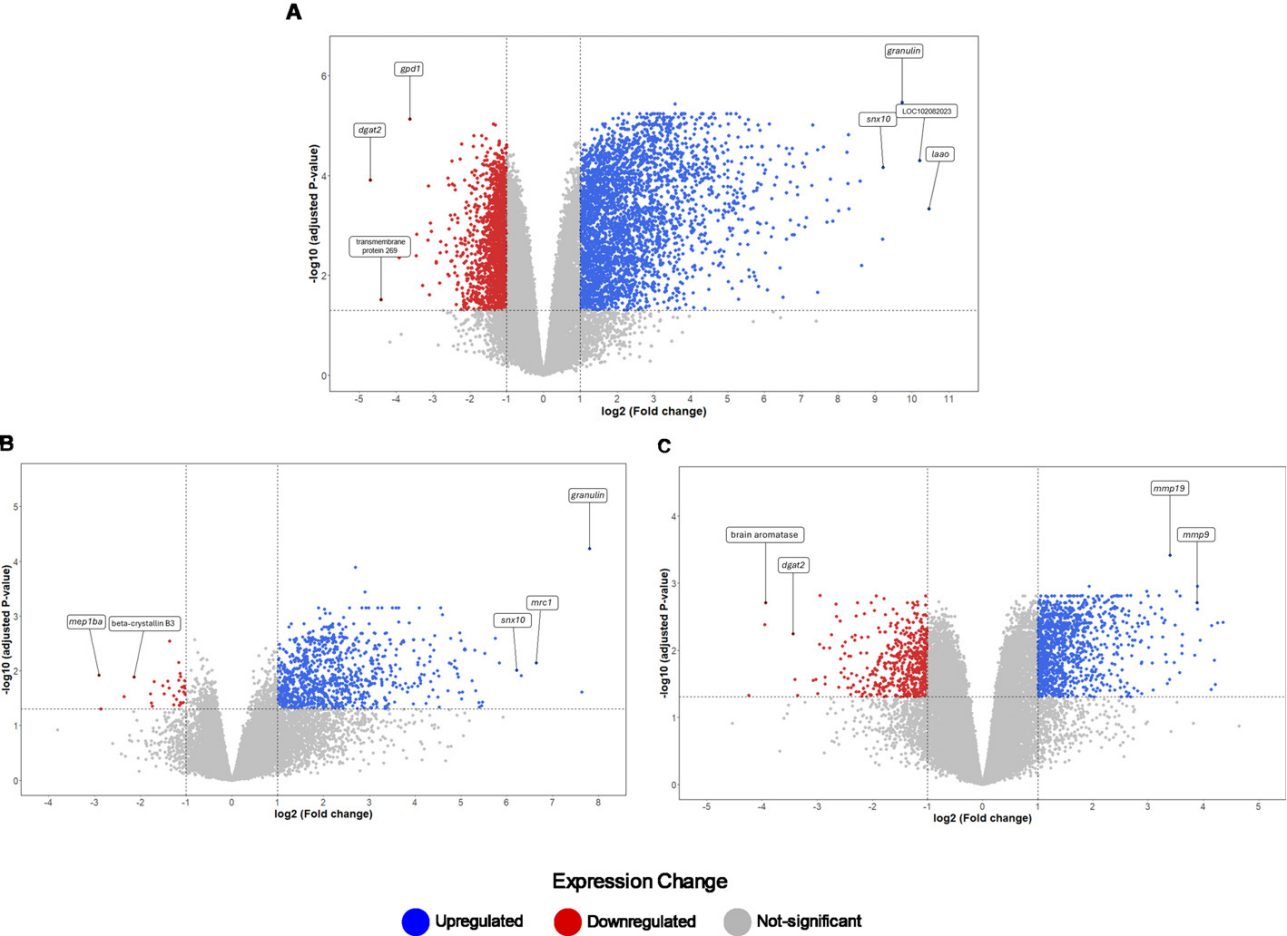
Distribution of DEGs based on fold changes for the Control, SA8-UEL, and SA10-UEL groups. **(A)** Volcano plot of DEGs identified between SA8-UEL exposed group and Control. **(B)** Volcano plot of DEGs identified between SA10-UEL exposed group and Control. **(C)** Volcano plot of DEGs identified between SA8-UEL exposed group and SA10-UEL exposed group. Genes represented in the plots correspond to specific LOC identifiers, including transmembrane protein 269 (LOC100689985), *laao* (LOC100703315), *mep1ba* (LOC100706112), beta-crystallin B3 (LOC100707280), and brain aromatase (LOC100534396). LOC102082023 is currently uncharacterized.

The analysis of global transcriptomic changes in the brain of Nile tilapia exposed to the SA8-UEL strain of *S. agalactiae* in comparison to the control group revealed 5,028 significantly differentially expressed genes (DEGs, |Log2 fold change| ≥ 1, Benjamini-Hochberg adjusted p-value ≤ 0.05) (3,281 upregulated and 1,747 downregulated), and a total of 997 DEGs in the SA10-UEL strain exposed group (965 upregulated and 32 downregulated). Also, 1,752 genes were differentially expressed (1,182 upregulated and 570 downregulated) between SA8-UEL and SA10-UEL exposed groups (**Figures 2**, **3**, **Supplementary Data Sheet 1**).

**Figure 4:**
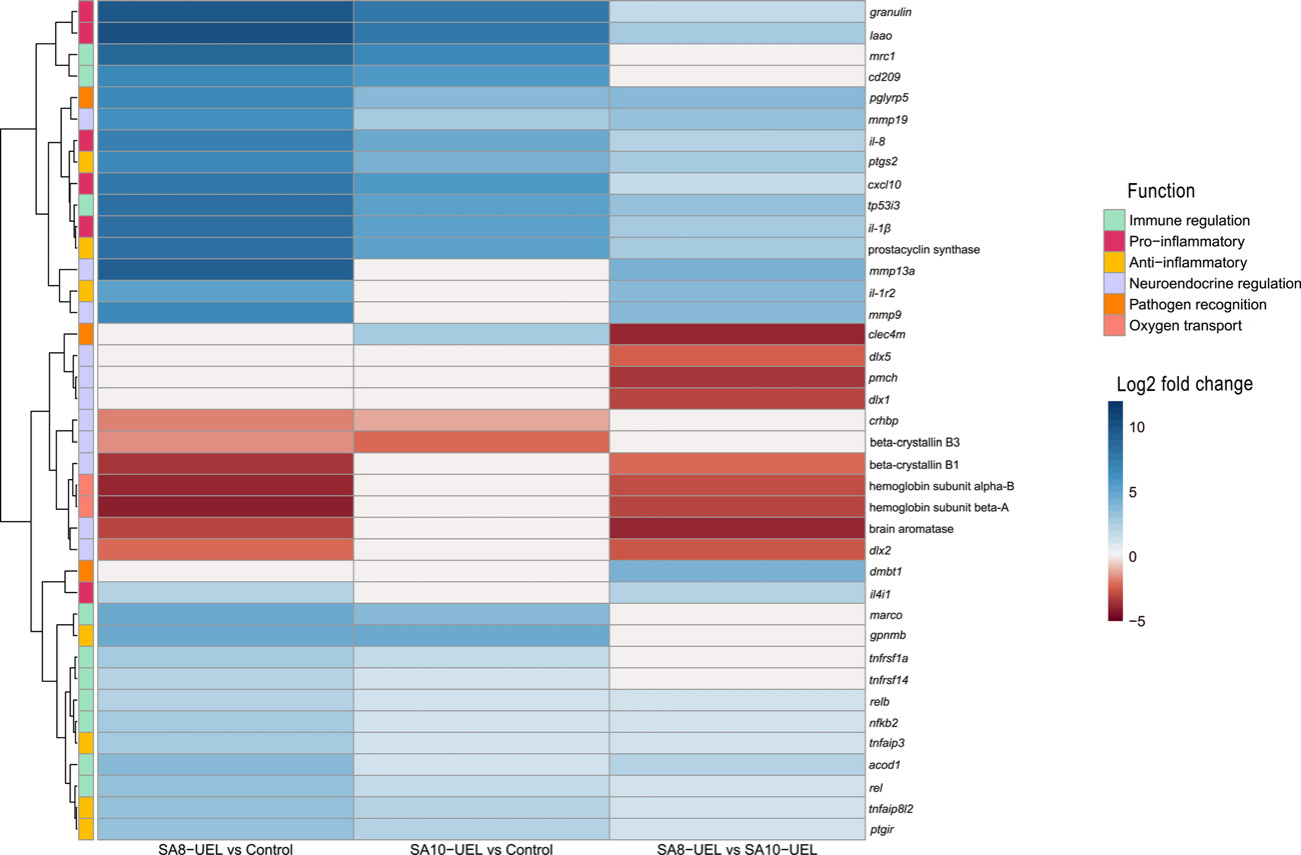
Heatmap of log2 fold change values for differentially expressed genes in Nile tilapia brains after infection with *Streptococcus agalactiae*. Rows represent genes, and columns represent pairwise comparisons between conditions (SA8-UEL vs Control, SA10-UEL vs Control, and SA8-UEL vs SA10-UEL). Colors indicate the magnitude and direction of fold change, with red shades indicating upregulation and blue shades indicating downregulation. Functional groups are annotated on the left. Comparisons with nonsignificant log2 fold change values were treated as 0. Genes represented in the plots correspond to specific LOC identifiers, including *laao* (LOC100703315), *mrc1* (LOC100710283), *cd209* (LOC1007062847), *cxcl10* (LOC100700788), *il1β* (LOC100707066), prostacyclin synthase (LOC100693506), *il1r2* (LOC100702124), beta-crystallin B1 (loc100707280), beta-crystallin B3 (LOC100705765), hemoglobin subunit alpha-B (LOC102080222), hemoglobin subunit beta-A (LOC100534396), dmbt1 (LOC102083301), and *il4i1* (LOC100697201).

Exposure to either *S. agalactiae* strain in Nile tilapia resulted in upregulation of immune and inflammation-related markers, including macrophage mannose receptor 1 (*MRC1*), *CD209* antigen, *granulin*, CXCL10, prostacyclin synthase, and interleukins *il-8*, *il-1β*, and il-4–induced 1 (IL4I1) (**Figure 4**, **Table 2**). Downregulation of genes involved in tissue repair (*beta-crystallin B1* and *B3*) and oxygen transport (hemoglobin subunit alpha-B and hemoglobin subunit beta-A) was also observed.

**Table 2:** Highly differentially expressed genes in Nile tilapia brain exposed to *S. agalactiae* strains SA8-UEL and SA10-UEL, with Log2 fold change (p-adj < 0.05) and corresponding standard deviation (SD).

Gene	Description	Log2 fold change (± SD)		
		SA8-UEL vs Control	SA10-UEL vs Control	SA8-UEL vs SA10-UEL
*rel*	v-rel avian reticuloendotheliosis viral oncogene homolog	3.09 (± 0.19)	1.82 (± 0.20)	1.26 (± 0.16)
*relb*	v-rel avian reticuloendotheliosis viral oncogene homolog B	2.15 (± 0.19)	1.08 (± 0.19)	1.07 (± 0.16)
*nfkb2*	nuclear factor of kappa light polypeptide gene enhancer in B-cells 2	2.83 (± 0.17)	1.41 (± 0.17)	1.43 (± 0.16)
LOC100700788	C-X-C motif chemokine 10	7.59 (± 0.41)	5.74 (± 0.41)	1.85 (± 0.16)
*il-8*	interleukin-8	7.08 (± 0.50)	4.90 (± 0.52)	2.18 (± 0.20)
*granulin*	progranulin	9.73 (± 0.28)	7.80 (± 0.28)	1.92 (± 0.17)
LOC100707066	interleukin-1β	8.01 (± 0.43)	5.40 (± 0.43)	2.62 (± 0.16)
*tnfrsf1a*	tumor necrosis factor receptor superfamily, member 1a	2.73 (± 0.27)	1.86 (± 0.28)	–
*tnfrsf14*	tumor necrosis factor receptor superfamily, member 14	2.43 (± 0.24)	1.46 (± 0.24)	–
LOC100710283	macrophage mannose receptor 1	8.59 (± 0.44)	6.64 (± 0.44)	–
LOC100706287	CD209 antigen	6.89 (± 0.46)	5.84 (± 0.46)	–
*marco*	macrophage receptor with collagenous structure	4.64 (± 0.28)	3.87 (± 0.28)	–
*tnfaip3*	tumor necrosis factor-α-induced proteins 3	2.54 (± 0.18)	1.12 (± 0.18)	1.41 (± 0.16)
*tnfaip8l2*	tumor necrosis factor-α-induced proteins 8-like 2	3.34 (± 0.23)	2.22 (± 0.24)	1.12 (± 0.16)
*ptgs2*	prostaglandin-endoperoxide synthase 2	6.88 (± 0.47)	4.30 (± 0.49)	2.59 (± 0.20)
*ptgir*	prostaglandin I2 receptor	3.47 (± 0.35)	2.19 (± 0.37)	1.28 (± 0.21)
LOC100693506	prostacyclin synthase	8.29 (± 0.57)	5.40 (± 0.59)	2.90 (± 0.21)
*gpnmb*	glycoprotein nmb	4.80 (± 0.33)	4.72 (± 0.33)	–
*crhbp*	corticotropin-releasing hormone binding protein	-1.81 (± 0.23)	-1.15 (± 0.21)	–
LOC100705765	beta-crystallin B1	-3.44 (± 0.45)	–	-2.24 (± 0.47)
LOC100707280	beta-crystallin B3	-1.46 (± 0.66)	-2.14 (± 0.69)	–
LOC102083301	deleted in malignant brain tumors 1 protein	–	–	4.21 (± 0.32)
*pglyrp5*	peptidoglycan recognition protein 5	6.76 (± 0.57)	3.95 (± 0.64)	3.95 (± 0.34)
LOC102079007	C-type lectin domain family 4 member M-like	–	2.57 (± 0.47)	-3.95 (± 0.52)
LOC100703315	L-amino-acid oxidase	10.46 (± 0.54)	7.64 (± 0.53)	2.83 (± 0.16)
LOC100697201	interleukin 4–induced 1	2.02 (± 0.44)	–	2.30 (± 0.42)
*tp53i3*	tumor protein p53 inducible protein 3	8.27 (± 0.35)	5.08 (± 0.35)	3.19 (± 0.16)
*acod1*	aconitate decarboxylase 1	3.64 (± 0.18)	1.26 (± 0.17)	2.38 (± 0.16)
LOC100702124	interleukin-1 receptor type 2	5.17 (± 0.37)	–	3.89 (± 0.27)
*mmp13a*	matrix metallopeptidase 13a	9.20 (± 0.59)	–	4.15 (± 0.23)
*mmp9*	matrix metallopeptidase 9	6.78 (± 0.25)	–	3.89 (± 0.16)
*mmp19*	matrix metallopeptidase 19	6.13 (± 0.37)	2.74 (± 0.39)	3.39 (± 0.20)
LOC100534396	brain aromatase	-3.07 (± 0.21)	–	-3.93 (± 0.21)
*pmch*	pro-melanin-concentrating hormone	–	–	-3.36 (± 0.27)
*dlx1*	distal-less homeobox 1	–	–	-3.00 (± 0.33)
*dlx2*	distal-less homeobox 2	-2.15 (± 0.45)	–	-2.57 (± 0.42)
*dlx5*	distal-less homeobox 5	–	–	-2.45 (± 0.37)
LOC102080222	hemoglobin subunit alpha-B	-3.91 (± 0.17)	–	-2.86 (± 0.16)
LOC100704059	hemoglobin subunit beta-A	-4.09 (± 0.17)	–	-3.03 (± 0.16)

The comparison between Nile tilapia exposed to the SA8-UEL strain with those exposed to the SA10-UEL strain revealed distinct patterns in the expression of pathogen recognition-related genes. Specifically, *LOC102083301*, coding deleted in malignant brain tumors 1 protein and *pglypr5* were upregulated in response to SA8-UEL, while *LOC102079007*, coding C-type lectin domain family 4 member M-like was downregulated. Furthermore, inflammatory markers, such as L-amino-acid oxidase (LAAO), IL4I1, *ptgs2*, *tp53i3*, and *acod1*, were also upregulated.

Some genes involved in neuroplasticity were also differentially expressed between the two strains. Among these, the ones coding matrix metallopeptidases 13a, 9, and 19 were upregulated, while brain aromatase and pro-melanin-concentrating hormone (*pmch*) were downregulated in response to SA8-UEL compared to the SA10-UEL strain.

### 3.3 Gene ontology enrichment and pathways analysis

After sorting, 4,979 DEGs (39% of the total) were categorized into 499 functional groups of three major categories (molecular function, biological process, cellular component) and 40 pathways (**Supplementary Data Sheet 2**). No biological pathways were enriched in the analysis of downregulated DEGS in the SA10-UEL strain exposed groups.

**Figure 5:**
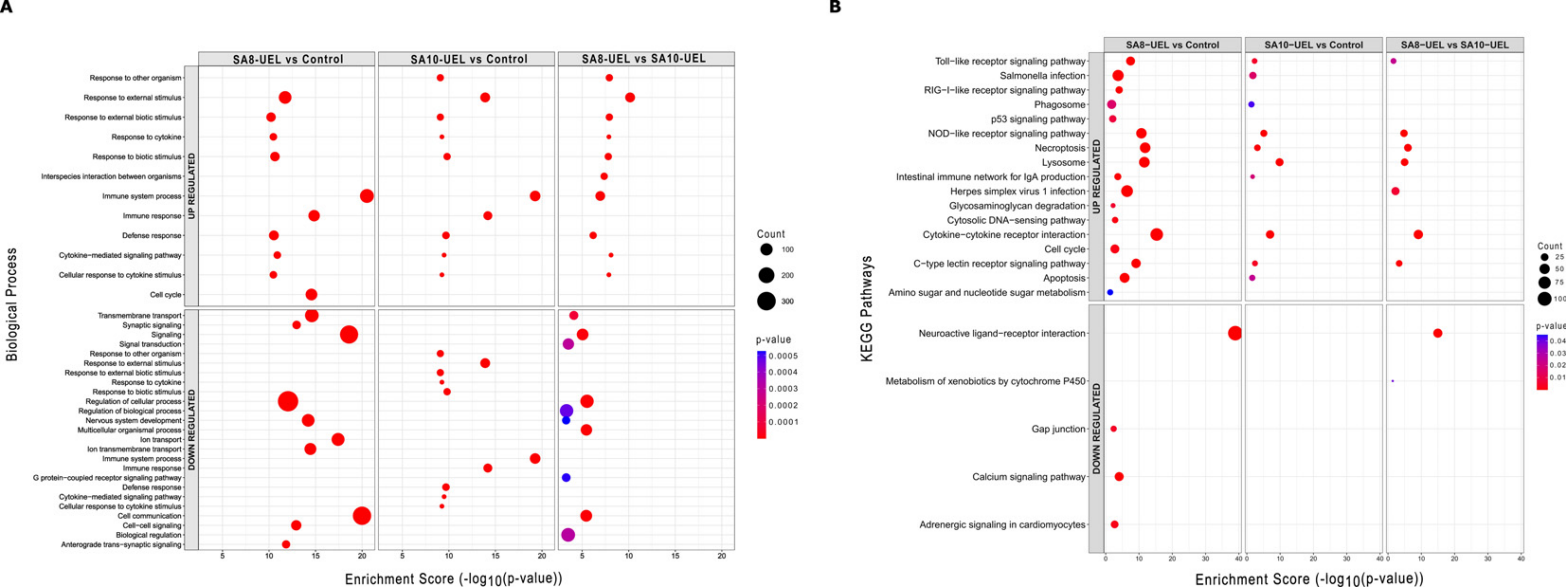
Gene Ontology (GO) biological processes and KEGG pathway enrichment analysis of differentially expressed genes in Nile tilapia brain following exposure to *Streptococcus agalactiae* strains SA8-UEL and SA10-UEL. The x-axis represents the enrichment score (−log_10_​(p-value)), while the size of the circles represents the number of DEGs in each category, and colors indicate statistical significance (p-value). **(A)** Top 10 enriched GO biological processes for upregulated and downregulated DEGs. **(B)** KEGG pathway enrichment analysis for upregulated and downregulated DEGs.

The differentially upregulated genes in the exposed groups were enriched in terms associated with cytokine receptors interaction, lysosome, and pathogens infection, whereas genes downregulated in the exposed groups caused the significant enrichment of GO terms related to signaling and cell communication. When comparing tilapia exposed to the SA8-UEL versus SA10-UEL strains, DEGs connected to immune and signaling receptors such as cytokines, NOD-like, and Toll-like were upregulated, whereas DEGs involved in signaling and neuroactive receptors were downregulated (**Figure 5**). The list of annotated DEGs and associated GO terms and pathways in both strains (SA8-UEL and SA10-UEL) compared to the control group and between each other is presented in **Supplementary Data Sheet 2**.

### 3.4 Validation of RNA-Seq profiles by qPCR

To validate the DEGs identified by the RNA-Seq, ten genes with different expression patterns were selected for qPCR confirmation. The fold changes from qPCR were compared to those obtained by RNA-Seq. Among the ten genes examined, eight (*LOC100710283*, *LOC100706287*, *pglyrp5*, *granulin*, *LOC100700788*, *il-8*, *LOC100707066*, and *LOC100703315*) showed consistent expression trends across both methods (**Supplementary Figure 1**). Although there were statistically significant differences in the fold changes between RNA-Seq and qPCR for some genes, the overall expression trends within the infected groups were similar between the two techniques. Specifically, despite variations in the magnitude of fold changes, the direction of expression (upregulation) and the relative amplitude of changes between groups observed in RNA-Seq were consistent with those identified by qPCR.

### 3.5 Confirmation of *S. agalactiae* brain infection by qPCR

The qPCR results confirmed the presence of bacterial RNA in the brain of exposed fish (**Supplementary Table 3**). Amplification of *rpoB* transcripts was detected in the SA8-UEL (mean Cq value of 35.11 ± 1.28), and SA10-UEL (mean Cq value of 36.56 ± 1.66) challenged groups. No bacterial RNA was detected in the control group samples.

## 4 Discussion

The present transcriptomics study focused on investigating the molecular responses in the brain of Nile tilapia following intraperitoneal injection of two serotypes of *S. agalactiae*. While this challenge approach does not mimic the natural infection route (e.g., mucosal surfaces), it was selected to ensure consistent bacterial dose delivery and bypass potential variability associated with natural infection routes. Our qPCR and transcriptomic data suggest that *S. agalactiae* breached the BBB, damaging the CNS through acute inflammation. Comparative analysis between pathogen-exposed and control groups revealed significant upregulation of genes associated with immune response and inflammation, alongside downregulation of genes related to neuroendocrine regulation and tissue repair.

Following exposure to *S. agalactiae*, we observed increased expression of *rel*, *relb*, and *nfkb2*, indicating activation of the nuclear factor κB (NF-κB) signaling cascade. This pathway is important for immune regulation and is known to induce the expression of pro-inflammatory mediators (43). Notably, two of the most relevant downstream targets of NF-κB, *CXCL10* and *il-8*, were also upregulated. These chemokines are associated with immune cell recruitment to infection sites. *CXCL10* has chemotactic activity towards T cells, macrophages, and natural killer cells (44, 45), while *il-8* recruits neutrophils (46). Notably, *CXCL10* has also been implicated in increased BBB permeability, which could facilitate immune cell migration into the brain (47).

In Mozambique tilapia, an alternatively spliced transcript of *granulin* modulates the expression of proinflammatory cytokines, including *il-1β* and *il-8* (48). In our study, the three genes—*granulin*, *il-1β*, and *il-8*—were upregulated, suggesting that *granulin* drives the expression of these cytokines. *il-1β* and *il-8* initiate innate immunity in vertebrates (49), mediate cellular communication, and trigger an inflammatory cascade (50).

The simultaneous upregulation of *il-1β*, *tnfrsf1a*, and *tnfrsf14* suggests BBB disruption, as *il-1β* is known to favor BBB plasticity and permeability, allowing immune cell infiltration into the CNS (51, 52). The *tnfrsf1a* and *tnfrsf14* genes encode receptors involved in tumor necrosis factor (TNF) signaling pathways (53). The expression of *tnfr1*, specifically, activates the NF-κB pathway (54), promoting inflammation and immune defense against pathogens (55, 56). TNF-α is a known contributor to BBB permeability and can lead to increased infiltration of immune cells into the CNS (57). Although TNF-α was not significantly upregulated, the expression of its receptors suggests that this factor may be active.

An existing hypothesis regarding *S. agalactiae* invasion into Nile tilapia’s CNS suggests that immune cells may function as “Trojan horses”, transporting the bacteria across the BBB (58). This idea is supported by the upregulated expression of macrophage mannose receptor 1 (*mrc1*), *CD209* antigen (DC-SIGN), and *marco* (coding for MARCO, macrophage receptor with collagenous structure). MRC1 and *CD209* are members of the C-type lectin superfamily, expressed in macrophages and dendritic cells (59, 60), while MARCO, a scavenger receptor, is expressed in certain macrophage subsets (61). Macrophages and dendritic cells, being phagocytic, can engulf *S. agalactiae* (62, 63), and inadvertently transport the bacteria into the CNS, contributing to pathogen dissemination.

The combined activation of the NF-κB pathway, TNF-related receptors, and recruitment of phagocytic cells, along with enrichment in cytokine-cytokine receptor interactions, indicates a pro-inflammatory response to *S. agalactiae* in the tilapia brain. While inflammation serves to contain the pathogen, prolonged activation can be harmful to the CNS. The upregulation of specific anti-inflammatory genes suggests compensatory mechanisms attempting to limit tissue damage during infection. One of the mechanisms involves the activation of *tnfaip3* and *tnfaip8l2*, encoding tumor necrosis factor-α-induced proteins 3 (A20) and 8-like 2 (TIPE2), respectively. Both are known as inhibitors of the NF-κB pathway (64, 65). Additionally, the activation of prostacyclin signaling, evidenced by *ptgs2*, *ptgir*, and *LOC100693506*, and the upregulation of *gpnmb* (glycoprotein nmb). PTGS2 (prostaglandin-endoperoxide synthase 2) has been shown to participate in the immune responses of common carp against *Aeromonas hydrophila* (66). The prostacyclin I2 receptor (*ptgir*) and prostacyclin synthase (*LOC100693506*) encode components important for anti-inflammatory effects, including vasodilation and prevention of platelet aggregation (67). Such as the PTG2, the glycoprotein nmb has an anti-inflammatory role and promotes disease resolution (68). Despite these regulatory mechanisms, the immune response remains skewed toward inflammation, as evidenced by the expression of multiple pro-inflammatory mediators.

The downregulation of *crhbp* (corticotropin-releasing hormone binding protein) implies a disruption in neuroendocrine regulation, specifically regarding stress. Crhbp modulates the availability of corticotropin-releasing hormone (CRH), essential for stress and behavioral responses in fish, as part of the hypothalamic-pituitary-interrenal (HPI) axis (69). Similarly, the downregulation of beta-crystallin genes (*LOC100705765* and *LOC100707280*), involved in preserving the transparency and refractive index of the eye lens (70), may explain the corneal opacity visible in fish with streptococcosis.

Comparing the results between SA8-UEL and SA10-UEL strains-exposed animals, we observed divergent expressions in pathogen recognition-related genes. *LOC102083301*, encoding Deleted in malignant brain tumors 1 (Dmbt1) protein, and *pglyrp5*, a gene in the peptidoglycan recognition protein (PGRP) family, were upregulated in response to SA8-UEL. This result suggests the potential for each *S. agalactiae* strain to interact differently with the host’s immune system, engaging different pathogen recognition pathways and consequently immune response initiation. In Nile tilapia, PGRP-SC, a member of the PGRP family, already demonstrated Zn^2+^-dependent peptidoglycan-degrading activity against *S. agalactiae* (71). Similarly, the Dmbt1 protein was already found to be upregulated in cells in response to proinflammatory stimuli and interacting with streptococcal cell wall adhesins (72). This upregulation suggests an immune recognition response that facilitates bacterial cell wall degradation and inflammation in SA8-UEL-infected fish. In contrast, the downregulation of the gene that encodes Clec4m (C-type lectin domain family 4 member M-like) suggests a different recognition mechanism for the SA10-UEL strain. Previous studies on Mincle, another C-type lectin receptor, characterized its ability to recognize different serotypes of *Streptococcus pneumoniae*, but with a limited role in bacterial phagocytosis and cytokine production (73). This finding suggests that Clec4m may recognize SA10-UEL without directly promoting immune activation, possibly contributing to delayed pathogen clearance in SA10-UEL-exposed fish.

The faster immune activation in SA8-UEL-exposed fish, driven by Dmbt1 and PGRP5, seems to elicit a more intense response, as indicated by the upregulation of *tnfaip3*, *nfkb2*, and *il-8*, all components of the NF-κB signaling pathway. The activation of this pathway is associated with strong pro-inflammatory responses, typically associated with acute infection control (74, 75). Additional genes involved in inflammation, including L-amino-acid oxidase (LAAO), *LOC100697201* (IL4I1, Interleukin 4-induced 1), *tp53i3* (tumor protein p53 inducible protein 3), *acod1* (aconitate decarboxylase 1), *ptgs2* (prostaglandin-endoperoxide synthase 2), and *LOC100702124* (IL1R2, interleukin-1 receptor type 2) were also upregulated. LAAO and IL4I1 contribute to bacterial defense by producing hydrogen peroxide, a compound that has antibacterial and pro-inflammatory effects (76–78). The activation of immune response is indicated by upregulation of *tp53i3* and *acod1*. *tp53i3* is associated with cellular stress responses and DNA repair; with dual roles promoting DNA repair under moderate stress, or inducing apoptosis when damage is irreparable (79, 80). The expression of *acod1* has already been proven to be important in anti-bacterial immunity in fish macrophages following lipopolysaccharide (LPS) stimulation (81–83). Moreover, the upregulation of *ptgs2* and IL1R2 shows a compensatory movement to counterbalance intense inflammation. IL1R2 is a decoy receptor and may help prevent excessive IL-1-driven signaling (84).

In response to SA8-UEL, metallopeptidases *mmp13a*, *mmp9*, and *mmp19*, which are involved in matrix remodeling and tissue repair, were also upregulated. In mammals, MMP-13 can activate MMP-9, recruiting immune cells and promoting wound healing (85, 86). MMP-19 may help regulate tissue integrity and limit damage by enhancing leukocyte infiltration, axonal regeneration, and astrogliosis for CNS recovery (87).

SA8-UEL exposure also appears to disrupt hormonal pathways in the Nile tilapia brain, as indicated by the downregulation of brain aromatase and *pmch* (pro-melanin concentrating hormone). Brain aromatase is involved in neurogenesis and neural repair (88, 89), while *PMCH* regulates appetite and behavior (90, 91). Reduced *pmch* expression correlates with the anorexigenic behavior observed in infected fish, suggesting that the SA8-UEL strain may intensify appetite suppression, likely as an adaptive response. Additionally, the downregulation of DLX genes family, particularly *dlx1*, *dlx2*, and *dlx5*. In zebrafish, the increased expression of *dlx* is associated with compensatory mechanisms for neuronal loss (92). The reduced expression of these genes in SA8-UEL-exposed fish may indicate more extensive tissue damage or a reduced regeneration capacity in comparison to SA10-UEL-exposed fish. Overall, SA8-UEL infection modulates genes that may lead to rapid immune activation, inflammation, and tissue repair. This shift is evident by the upregulation of genes involved in inflammation and tissue remodeling, along with the downregulation of genes involved in oxygen transport, neuroendocrine regulation, and behavioral responses. The faster immune response in SA8-UEL-exposed fish may explain the steady mortality rate, while the delayed response in SA10-UEL-exposed fish corresponds with acute mortality peaks within the initial days post-infection, and more severe CNS symptoms Furthermore, the SA10-UEL strain induced more pronounced neuroendocrine disruptions, likely contributing to the erratic behavior observed (**Supplementary Figure 2**).

During validation of the DEGs, 8 out of 10 genes analyzed showed consistent expression patterns across RNA-Seq and qPCR. Discrepancies in fold change were observed in two genes. *LOC102083301* was downregulated in the qPCR analysis and upregulated in the RNA-Seq results, and the *pmch* gene downregulated during RNA-Seq analysis and upregulated in qPCR in the SA8-UEL exposed group. These differences may be attributed to the use of samples from independent experiments or incorrect assignment of RNA-Seq reads to paralogous genes. Despite these minor inconsistencies, the qPCR validation supports the overall trends observed in the RNA-Seq data, indicating the reliability and accuracy of the differential expression analysis.

## 5 Conclusions

**Figure 6:**
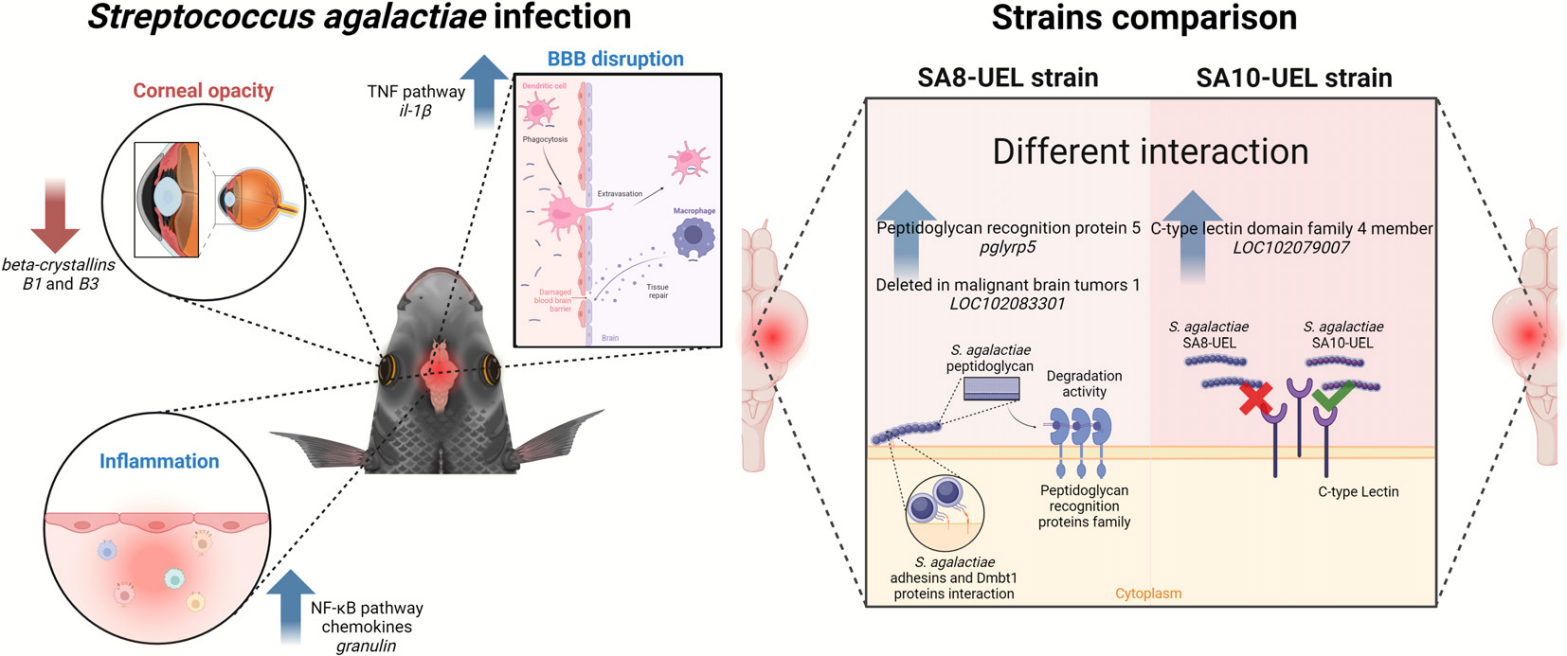
Overview of differential immune and physiological responses in Nile tilapia infected with two strains of *Streptococcus agalactiae* (SA8-UEL and SA10-UEL). Infection triggers inflammation, blood-brain barrier (BBB) disruption, and corneal opacity. BBB disruption permits immune cell infiltration into the brain, initiating tissue repair mechanisms but also allowing bacterial entry. Strain-specific host immune recognition responses are illustrated: SA8-UEL infection upregulates *pglyrp5* and *LOC102083301* (Deleted in Malignant Brain Tumors 1), promoting peptidoglycan degradation, while SA10-UEL infection upregulates *LOC102079007* (C-type lectin domain family 4 member), indicating distinct immune recognition pathways for each strain.

Our findings suggest that Nile tilapia exhibit distinct immune responses to *S. agalactiae* serotypes Ib and III, with serotype-specific gene expression profiles indicating differential interactions with the host’s immune and neuroendocrine pathways. A graphical summary of the main findings of this manuscript is depicted in **Figure 6**.

Despite the intraperitoneal route of infection, our findings indicate that *S. agalactiae* breached the BBB, initiating CNS inflammation. We identified significant upregulation of immune-related genes, including important components of the NF-κB pathway, alongside the downregulation of genes associated with tissue repair and neuroendocrine functions.

In fish infected with the SA8-UEL strain (serotype Ib), the upregulation of *pglyrp5* and the gene that encodes Dmbt1, both involved in bacterial cell wall recognition and degradation, indicates a more immediate immune response. In contrast, the SA10-UEL strain (serotype III) elicits a less pronounced immune activation, with the upregulation of the gene coding Clec4m. This C-type lectin receptor, although involved in bacterial recognition, does not directly trigger an immune response, potentially leading to insufficient pathogen clearance compared to serotype Ib. This differential response may explain the more severe CNS symptoms and behavioral disturbances, such as erratic swimming, observed in SA10-UEL-infected fish.

To the best of our knowledge, this is the first transcriptomic study to identify serotype-specific immune responses induced by two *S. agalactiae* strains in Nile tilapia. Understanding the immunological and neuroendocrine pathways activated by different bacterial serotypes provides important insights into host-pathogen interactions and can guide more targeted disease management strategies for the aquaculture industry.

## Data Availability

The datasets presented in this study can be found in online repositories. The names of the repository/repositories and accession number(s) can be found below: https://www.ncbi.nlm.nih.gov/, PRJNA1049341.
