# Supplementary material for: Comparative transcriptome analysis reveals a serotype-specific immune response in Nile tilapia (Oreochromis niloticus) infected with Streptococcus agalactiae
Source: Front Immunol. 2025 Jan 10;15:1528721. doi: 10.3389/fimmu.2024.1528721 (PMC11758187; doi:10.3389/fimmu.2024.1528721)
Supplement: Supplementary file 1 [file DataSheet1.pdf]

Supplementary Material

1 Supplementary Figures and Tables

Supplementary Table 1. Sampling time points for brain tissue collection after fish challenge in control and challenged groups.

Sampling time	Group	Sample ID
Day 7	Control	TIL01008
	SA8-UEL infected	TIL01111
Day 8	Control	TIL01009
	SA8-UEL infected	TIL01112
Day 9	SA8-UEL infected	TIL01113
Day 11	SA10-UEL infected	TIL01210
Day 12	Control	TIL01010
	SA8-UEL infected	TIL01114
	SA8-UEL infected	TIL01115
	SA10-UEL infected	TIL01212
	SA10-UEL infected	TIL01214
Day 13	Control	TIL01011
	SA10-UEL infected	TIL01215
	SA10-UEL infected	TIL01216

Supplementary Table 2. Details of primers used for expression analysis by qRT-PCR.

Gene name	Primer sequence	Melting temperature (ºC)	qPCR efficiency (%)	Amplicon size (bp)
LOC100710283	F- 5’ CAT TTG CCT GGA TGG ATG ATT C 3’<br>R- 5’ AGT AAT AGC CAC GCA GTT CTC 3’	60	95	98
LOC100706287	F- 5’ CAG CAA ATT TCA CTC TGC AGT TTA T 3’<br>R- 5’ ACA CCA GCC AAG AGG AGT AA 3’	60	98	99
LOC102083301	F- 5’ TCT CTC ATG TGC AGG TCA AC 3’<br>R- 5’ CAC ACC ACC TCA GCA TCT C 3’	58	99	141
pglyrp5	F- 5’ AGC CAG CCT TGT CAG TAT TC 3’<br>R- 5’ CAC TCG GCG TCT CAT TGT T 3’	60	106	200
granulin	F- 5’ GAT GTT GAG GAT CAC TCT GTG 3’<br>R- 5’ GAA CAA GTA CTC CCA TCA GGA C 3’	60	97	93
LOC100700788	F- 5’ ATC GGC GTG AAG TCT GAA ATA A 3’<br>R- 5’ CCA CTG TTG AAG GTA ACG AGA A 3’	60	92	92
il-8	F- 5’ CGC TTC AGG CTT CAT CTA CTT 3’<br>R- 5’ CCA CTC CCA TGC CTT CAC 3’	62	95	157
LOC100707066	F- 5’ GAG AGC ATT GTG GAA GAG CA 3’<br>R- 5’ TGC TGC TGT GGA GAA GAA C 3’	60	91	130
LOC100703315	F- 5’ GCA CAC TCC GCA GTT AGA G 3’<br>F- 5’ GAC AGC ACA TAA TTT CCG TTG G 3’	60	97	150
pmch	F- 5’ GCA GAG GGT GGT ATA ACA GAA G 3’<br>R- 5’ GCC TTG CAT CCG AGA TGA T 3’	60	90	151
ubce	F- 5’ CTC TCA AAT CAA TGC CAC TTC C 3’<br>R- 5’ CCC TGG TGG AGG TTC CTT GT 3’	59	91	130
eef1a	F- 5’ GCA CGC TCT GCT GGC CTT T 3’<br>R- 5’ GCG CTC AAT CTT CCA TCC C 3’	59	93	250
b2m	F- 5’ TTG GCT GTT ATT GCA GCT TTC T 3’<br>R- 5’ AAG TGC CAG TCC TGG TTG AAG 3’	60	92	224
tuba	F- 5’ AGC CAG ACG GAC AGA TGC C 3’<br>R- 5’ TTC CTG CAC GCA CCT CAT C 3’	60	96	153

Supplementary Table 3. Cq values and standard deviation for Control, SA8-UEL, and SA10-UEL groups in qPCR analysis for confirmation of *S. agalactiae* infection in the brain.

Sample	Mean Cq value duplicates	Mean Cq value group	Standard deviation
Control 1	-	-	-
Control 2	-		
Control 3	-		
Control 4	-		
Control 5	-		
Control 6	-		
Control 7	-		
SA8-UEL 1	33.80	35.11	1.28
SA8-UEL 2	33.13		
SA8-UEL 3	36.26		
SA8-UEL 4	33.74		
SA8-UEL 5	36.34		
SA8-UEL 6	37.59		
SA8-UEL 7	34.91		
SA10-UEL 1	36.21	36.56	1.66
SA10-UEL 2	36.26		
SA10-UEL 3	34.78		
SA10-UEL 4	36.84		
SA10-UEL 5	38.51		
SA10-UEL 6	37.80		
SA10-UEL 7	35.55		

1.1 Supplementary Figures

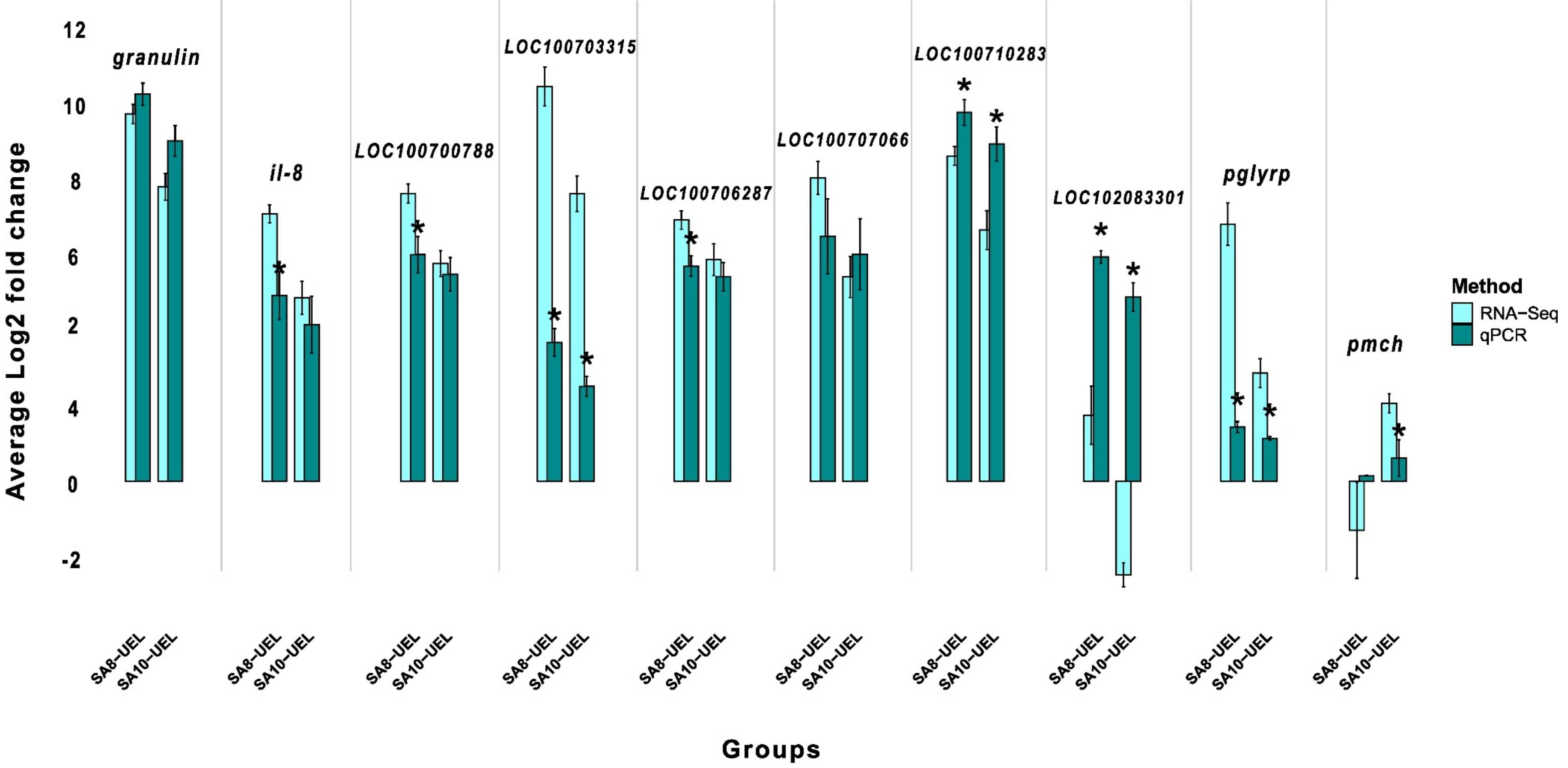

Supplementary Figure 1. Correlation between RNA-Seq normalized counts and relative gene expression from qPCR. Fold changes in gene expression were calculated relative to the control group and normalized using a geometric normalization factor derived from the housekeeping genes *tuba* and *ubce*. Data are presented as mean ± SE of Log2 fold changes (treatment groups/control). Statistical significance was assessed using the Mann-Whitney U Test (Wilcoxon rank-sum test) with $p < 0.05$, indicated by an asterisk (*).

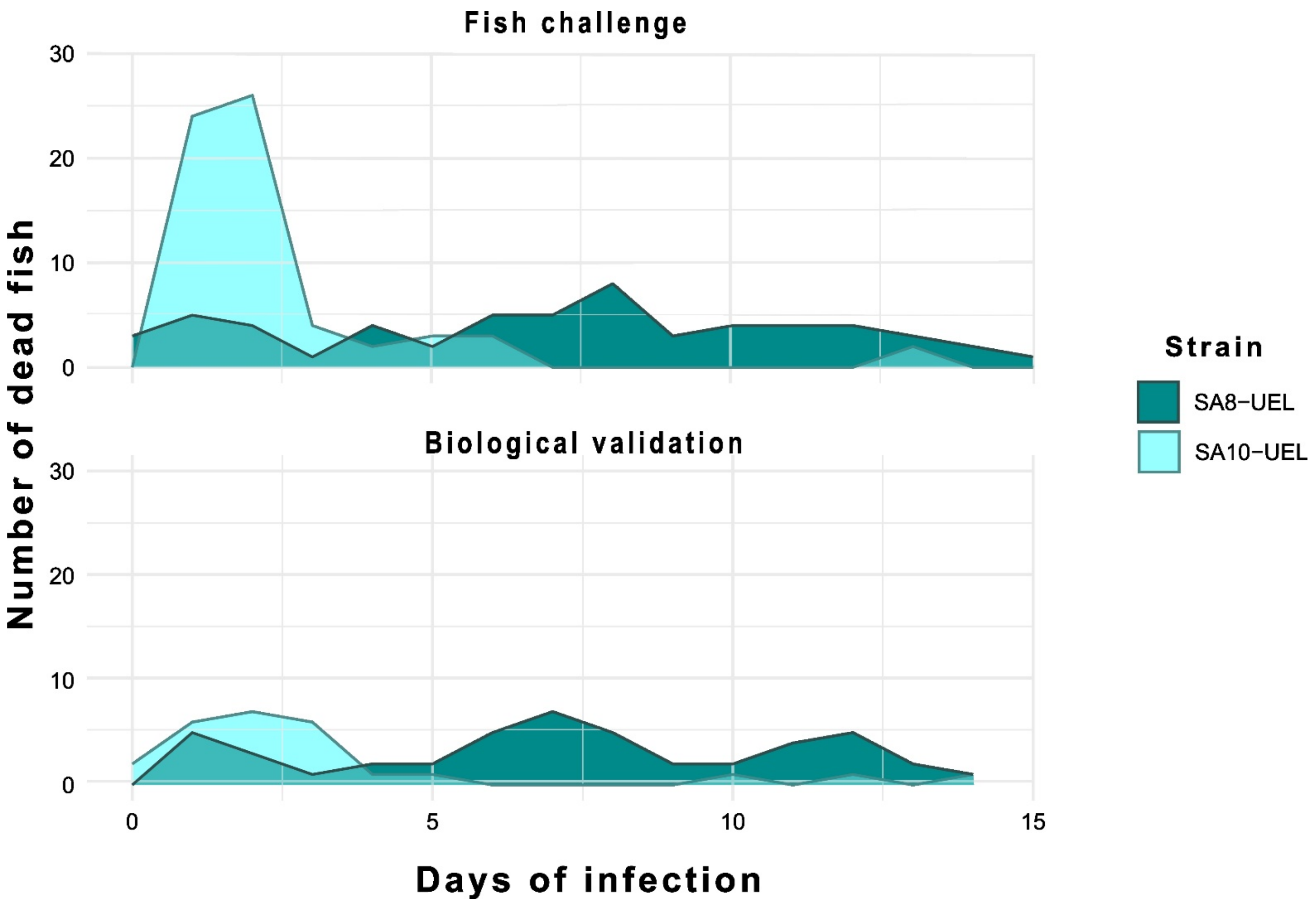

Supplementary Figure 2. Mortality patterns in Nile tilapia following infection with *S. agalactiae* strain SA8-UEL and SA10-UEL. Samples from the first experiment ("Fish challenge") were used for RNA-seq and the challenge was repeated ("Biological validation") for validation of gene expression data by qPCR.
